# The use of pyrocarbon as a bearing surface in shoulder arthroplasty: A systematic review and meta-analysis

**DOI:** 10.1177/17585732251345077

**Published:** 2025-05-27

**Authors:** Geoffrey W Schemitsch, Patrick Carroll, Patrick Henry, Diane Nam, Ujash Sheth

**Affiliations:** 1 Division of Orthopaedic Surgery, 7938 University of Toronto, Toronto, Ontario, Canada; 2 Department of Orthopaedic Surgery, Western University, London, Ontario, Canada; 3 Division of Orthopaedic Surgery, 71545 Sunnybrook Health Sciences Centre, Toronto, Ontario, Canada

**Keywords:** Shoulder arthroplasty, shoulder hemiarthroplasty, pyrocarbon, systematic review

## Abstract

**Background:**

Pyrocarbon has been trialed as an alternative bearing surface to metal for young active patients with glenohumeral joint arthritis. The aim of this study was to systematically review and summarize the available evidence on reported outcomes of pyrocarbon implants in shoulder arthroplasty.

**Methods:**

A systematic review was conducted according and reported according to standardized guidelines. Patient demographics, complications, implant survivorship and patient-reported outcome measures were extracted and included in the quantitative analysis. Outcome data was summarized with weighted mean differences and proportions.

**Results:**

Fifteen studies were included with 904 patients and a median follow-up of 38 months. The pooled mean range of motion improvement was 43.9 degrees (95% confidence interval [95% CI] 36.7–51.2) in forward elevation and 24.1 degrees (95% CI 18.4–29.9) in external rotation. Pooled mean Constant Score improvement was 34.7 (95% CI 29.4–40.1) and Subjective Shoulder Value improvement was 40.6 (95% CI 33.4–47.8). The overall pooled re-operation rate was 8.03% (95% CI 5%–12.7%).

**Discussion:**

Pyrocarbon shoulder implants demonstrated improvements in functional outcomes with low revision rates at early- to mid-term follow-up. Further well-designed prospective studies with long-term follow-up are required to verify the safety and efficacy of pyrocarbon implants in shoulder arthroplasty.

## Introduction

Glenohumeral osteoarthritis (GHO) is a debilitating condition characterized by pain, loss of shoulder function and disability. While osteoarthritis typically effects elderly populations, a reported 17–19% of patients presenting with GHO are <55 years-old.^1,2^ The initial treatment of GHO is characterized by non-operative management strategies including physical therapy, anti-inflammatory medications, joint injections and activity modification. Failure of non-operative management prompts consideration of surgical management.

The surgical management of GHO in young active patients presents additional challenges and considerations when compared with elderly patients. In particular, young patients generally have higher post-operative expectations, greater functional demands and present with more complex secondary causes of GHO including post-traumatic arthritis, inflammatory arthritis and avascular necrosis.^1–4^ With the expectation of future revision arthroplasty in young patients, implant survivorship, preservation of bone stock and relative ease of revision arthroplasty are other key considerations in treatment planning.

Surgical treatment options in young patients with GHO include total shoulder arthroplasty (TSA), hemiarthroplasty and glenoid resurfacing. The use of TSA has demonstrated favourable restoration of function and pain relief.^5,6^ However, TSA has been associated with glenoid loosening necessitating revision surgery.^5,7,8^ This complication is particularly problematic in young active patients. Hemiarthroplasty is often suggested as a means of avoiding glenoid loosening to preserve bone stock in anticipation of future revision surgeries. However, when compared with TSA, hemiarthroplasty has demonstrated lower levels of patient satisfaction and can lead to glenoid erosion requiring revision to TSA.^5,7,9^ Recently, an alternate weight-bearing surface, pyrocarbon, has been trialed with the goal of decreasing rates of symptomatic glenoid erosion. Traditional hemiarthroplasty implants coated with pyrocarbon are most commonly utilized. Pyrocarbon interposition shoulder arthroplasty (PISA) is also available and utilizes a pyrocarbon-coated humeral bearing surface with a graphite core that is positioned within a reamed cavity within the proximal humerus.^10^ The pyrocarbon implant articulates with the native glenoid. Clinically, pyrocarbon has demonstrated positive outcomes as a bearing surface in hand and wrist interposition and hemiarthroplasty.^11–13^ In comparison, the use of pyrocarbon in shoulder arthroplasty is relatively novel. The aim of the present study was to systematically review the best available evidence and provide insight into the reported outcomes of pyrocarbon implants in shoulder arthroplasty.

## Methods

**Figure 1.**
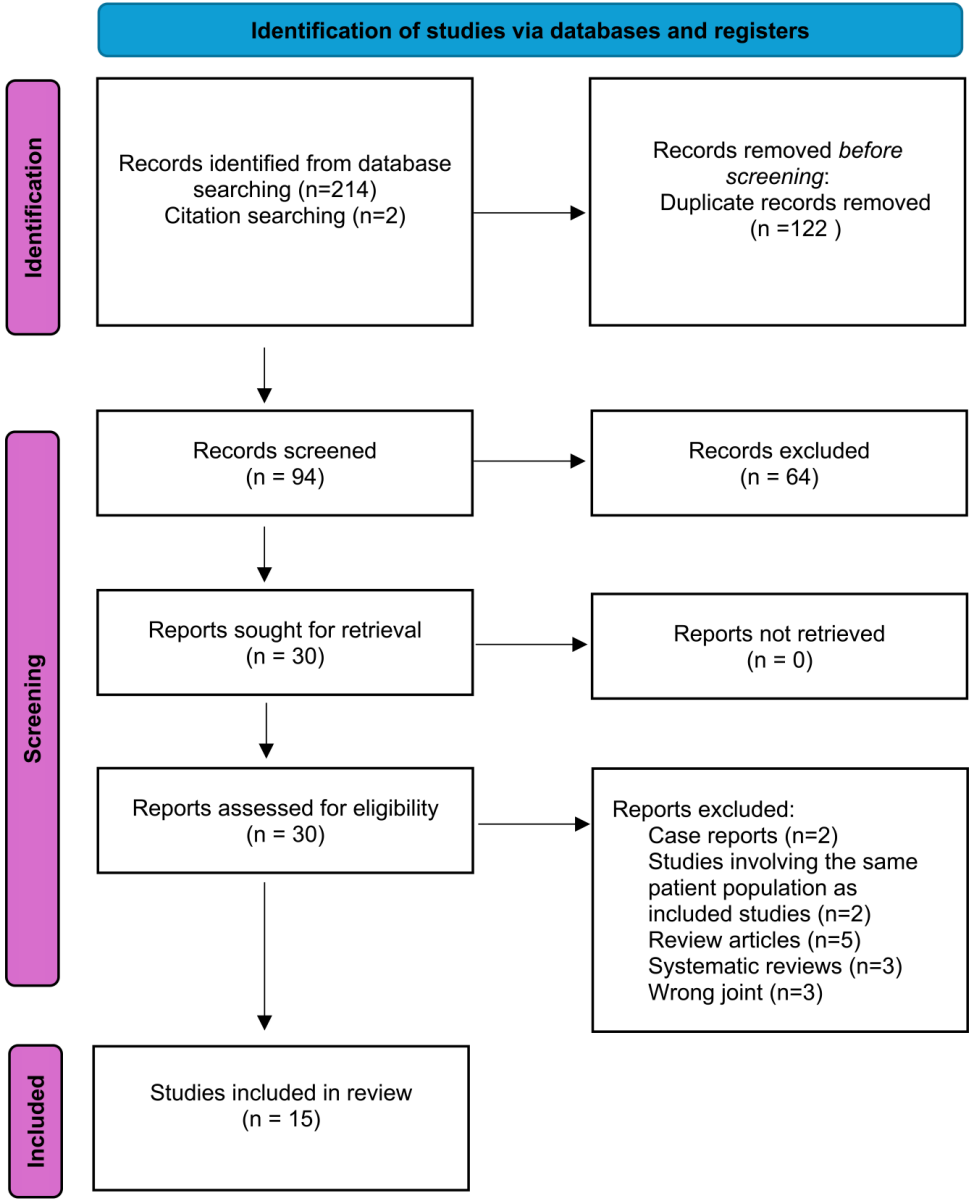
Preferred reporting items for systematic reviews and meta-analyses (PRISMA) flow diagram.

This systematic review was conducted according to the methods of the Cochrane Handbook for Systematic Reviews and is reported according to the Preferred Reporting Items for Systematic Reviews and Meta-Analyses guidelines (Figure 1).^14,15^

### Search strategy and eligibility criteria

The search was created with assistance from a health sciences librarian with expertise in conducting systematic reviews. The search was performed using Medline, Embase, CENTRAL and Scopus. These databases were screened from their inception until 6^th^ January 2023 (Table 1). Duplicates were removed via DOI deduplication and the Bramer method.^16^ The Boolean search terms were: ‘shoulder joint’ or ‘shoulder’ or glenohumeral’ or ‘glenoid fossa’ or ‘scapula’ or ‘humerus’ or ‘humeral’ or ‘arthroplasty’ or ‘hemiarthroplasty’ or ‘orthopaedic procedures’ or ‘joint prosthesis’ or ‘metal-on-metal joint prosthesis’ or ‘bone-implant interface’ or ‘prostheses and implants’ or ‘shoulder arthroplasty’ or ‘shoulder replacement’ or ‘shoulder prosthesis’ or ‘pyrocarbon’ or ‘pyrolytic carbon’ or ‘pyrocardan’.

**Table 1.** Summary of search results.

Database	Results (database exports from 6 January 2023)	Results (deduplicated via Bramer Method)
Medline	66	66
Embase	73	71
Central	2	2
Scopus	73	72
Total	214	211
Duplicates removed via DOI deduplication and Bramer Method	n/a	119
Total (duplicated)	n/a	92

### Study selection

Two individuals (EM and PC) independently conducted a computerized search of the electronic databases Medline, Embase, CENTRAL and Scopus (1). Two authors (GS and PC) reviewed the titles and abstracts of the 92 articles identified in the search; 15 articles were deemed suitable for the study.^8,17–29^ There was no disagreement between the reviewers, and a third reviewer was not necessary.

### Assessment of eligibility

We included all studies in which patients received a pyrocarbon shoulder arthroplasty procedure for any cause. Studies written in all languages were considered. Exclusion criteria included: (a) population: studies evaluating pediatric patients (age less than 18 years old), patients treated with other bearing surfaces; (b) study designs: review articles, animal studies, surveys, case reports, editorials and commentaries; (c) publication status: unpublished works were not considered. Where available patient demographics, complications, implant survivorship and patient-reported outcome measures were included in the quantitative analysis.

### Data extraction

Data extraction was completed using an Excel sheet template. Data that was extracted included authors, publication year, study level, study type, demographic data (age, sex, sample size), type of arthroplasty, reason for index operation, follow-up time, functional outcomes as measured by the Constant Score or Subjective Should Value (SSV) and range of motion in forward elevation and external rotation measured in degrees. Re-operation was defined as an unplanned return to the operating room for any cause. Reasons for reoperation were obtained when available.

### Statistical analysis

Statistical analyses were completed using R Statistical Software (*R Foundation for Statistical Computing, Vienna, Austria*). Data was pooled using a random effects model using inverse of variance weights. *T*^2^ was calculated using the restricted maximum likelihood method. Confidence intervals for pooled estimates were calculated using the Hartung–Knapp adjustment.^30^ For continuous variables (range of motion, SSV, Constant Score), study sample size, mean and standard deviation (SD) were used to calculate mean differences between pre-operative and post-operative mean values, quantifying the degree of post-operative improvement. Medians and interquartile ranges were converted to means and SDs, respectively, assuming a normal distribution.^31^ In one study where no measure of variance was reported, the mean variance from all other included studies was inputted for the missing data.^26^ The pooled proportion of patients who required a re-operation was calculated using study-specific logit-transformed proportions inputted into a generalized linear mixed-effects model.

Subgroup analyses were completed comparing summary pooled estimates stratified by treatment type (hemiarthroplasty, PISA and hemi-resurfacing). Subgroup differences were tested statistically using a Q-test assuming a type I error of 0.05.

Heterogeneity was assessed using the *I*^2^ statistic, *T*^2^ and prediction intervals. The *I*^2^ statistic estimated the proportion of heterogeneity between included studies that could attributed to differences in true effects versus differences due to sampling error.^32^ *T*^2^ was calculated to estimate the magnitude of variance of true effects between studies on a log scale and was used to calculate prediction intervals.^33^ Prediction intervals were calculated to predict the range of summary effects at 95% confidence for future studies.^34^

### Assessment of quality

Quality assessment was performed using the Methodological Index for Non-Randomized Studies (MINORS), a validated scoring system for the methodologic quality of comparative and non-comparative non-randomized surgical studies.^35^ The scoring categories are assigned a rating from 0 to 2, where the ideal score for a comparative study is 24. Categorization of the quality of the MINORS score was based on previous systematic reviews that separated the MINORS into: very low: 0 < MINORS score < 6; low: 6 ≤ MINORS score < 10; fair: 10 ≤ MINORS score ≤ 14; and good: MINORS score >14.^36,37^ Risk of bias assessment was completed by one reviewer.

## Results

Fifteen studies were included in the final analysis (Figure 1). Three studies were level III evidence using large database retrospective cohort designs and 12 were level IV evidence with 7 retrospective and 5 prospective case series designs. Ten studies evaluated pyrocarbon hemiarthroplasty implants,^18–22,25–28,38^ four studies evaluated PISA implants^8,17,23,24^ and one study evaluated pyrocarbon hemi-resurfacing.^29^ A total of 904 patients were included. The median of the reported mean ages was 53 years. The most common indications for surgery were primary osteoarthritis, osteonecrosis, fracture sequelae and instability. The median of the reported follow-up intervals was 38 months. MINORS score was low in two studies, fair in 11 studies and good in two studies. Study demographics are summarized in Table 2.

**Table 2.** Study demographics.

Author & year	Study location	Journal	Study level	Study design	Patients	Sex (% male)	Age (yrs.)	Diagnosis	Mean follow-up (months)	MINORS score
Barret et al., 2019	Nice, France	JSES	4	Prospective Case Series	56	53.50%	52 (39–65)	Primary OA 18 Fracture sequela 16 Instability arthropathy 15 Hemi revision 4 ON 3 RA 2	47	15
Barret et al., 2021	Toulouse, France	JSES	4	Retrospective Case Series	30	53.30%	54 ± 10	ON 12 OA 10 Fracture 4 Failed humeral head resurfacing 4	38	13
Caughey et al. 2023	Auckland, New Zealand	JSES	4	Retrospective Case Series	58	56.90%	n/a	Primary OA 40 Post-instability 9 Fracture sequelae 5 AVN 3 Synovial osteochrondromatosis 1	62	9
Cointat et al., 2022	Nice, France	JSES	4	Retrospective Case Series	62	68.80%	53 (25–60)	Primary OA 20 Post traumatic OA 13 Instability arthritis 15 ON 13 RA 3	33	12
Garret et al., 2022	Lyon, France	JSES International	4	Prospective Case Series	67	45.80%	50 ± 12	Primary OA 42 AVN 13 Secondary OA post instability or fracture 12	67.6 ± 9.3	12
Garret et al., 2019	Lyon, France	JSES Open Access	4	Prospective Case Series	65	67.20%	57.9 ± 13.3	Primary OA 37 ON 11 Secondary OA 11 (7 instability, 4 fracture) RA 2	25.9 ± 3.3	12
Gao et al., 2023	Auckland, New Zealand	JSES	3	Retrospective Cohort	159	66%	53 ± 11.2	Primary OA 76 AVN 33 Fracture sequelae 27 RA 5 Other 18	39.6	17
Hirakawa et al., 2021	St Grgoire, France	JSES	4	Retrospective Case Series	10	n/a	55.2	Primary OA 7 AVN 1 Fracture sequelae 1 Instability 1	48.5 (24–84)	8
Hudek et al., 2017	Germany	Orthopade	4	Retrospective Case Series	10	40%	55.6 ± 12.9	Failed proximal humerus ORIF 10	42.8 (±15)	13
Kany et al., 2021	Saint-Jean, France	Orthopaedics & Traumatology: Surgery & Research	4	Retrospective Cohort	24	66.70%	44 (35–50)	Primary OA 12 Instability 10 Other 2	36 (24–77)	11
Khoriati et al., 2023	Australia	JSES	3	Retrospective Cohort	159	85.5%	46.4 ± 6.4	n/a	31.2 ± 24	13
Kleim et al., 2021	Bavaria, Germany	Archives of Orthopaedic Trauma and Surgery	4	Prospective Case Series	21	81%	58.4 (22–84)	Primary OA 14 Fracture sequelae 1 AVN 3 Instability 3	n/a	11
McBride et al., 2022	Southport, Australia	JSES	3	Retrospective Cohort	163	85.30%	n/a	n/a	n/a	14
Novi et al., 2022	Modena, Italy	Musculoskeletal Surgery	4	Retrospective Case Series	4	n/a	23 (19–30)	Locked anterior shoulder dislocation 4	36	10
Tsitlakidis et al., 2021	Heidelberg, Germany	Orthopaedics & Traumatology: Surgery & Research	4	Prospective Cohort	16	43.80%	52.8 ± 10.8	Primary OA 10 AVN 4 RA 2	24.3 ± 8.1	14

AVN: avascular necrosis; M: male; JSES: Journal of Shoulder and Elbow Surgery; OA: osteoarthritis; ON: osteonecrosis; ORIF: open reduction internal fixation; RA: rheumatoid arthritis, n/a: not available or described.

### Functional outcomes

Seven studies reported pre-operative and post-operative range of motion values.^8,20,22,23,25–27^ The mean difference between pre-operative and post-operative range of motion was 43.9 (95% CI 36.7–51.2) degrees in forward elevation and 24.1 (95% CI 18.4–29.9) degrees in external rotation. When stratified by type of implant, there was no statistically significant difference in range of motion between hemiarthroplasty and PISA implants in forward elevation (hemiarthroplasty 43.8 degrees; 95% CI 30.3–57.3 vs. PISA 43.4 degrees; 95% CI 21.3–65.5, *p* = 0.954) and in external rotation (hemiarthroplasty 26.5 degrees; 95% CI 25.4–27.6 vs. PISA 19.6 degrees; 95% CI −6.3 to 45.5 degrees, *p* = 0.252). Constant Score was measured in nine studies.^8,17,18,20,22,23,25–27^ Mean Constant Score improvement was 34.7 (95% CI 29.4–40.1). There was no statistically significant difference in Constant Score improvement between hemiarthroplasty (mean improvement 36.1; 95% CI 28–44.2) and PISA (mean improvement 33; 95% CI 25.7–40.4) (*p* = 0.397). Pre-operative and post-operative SSV was measured in five studies.^8,20–23,25,26^ Mean SSV score improvement was 40.6 (95% CI 33.4–47.8). There was no statistically significant difference between hemiarthroplasty (mean improvement 41.7; 95% CI 24.4–59.1) versus PISA (mean improvement 38.7; 95% CI 20.8–56.6) (*p* = 0.473). Functional outcome scores are summarized in Table 3.

**Table 3.** Pooled outcome data with measures of heterogeneity and subgroup analysis stratified by implant type.

Measure	Sample	Pooled results (95% CI)	Heterogeneity (95% CI)	Subgroup result (95% CI)	*p* value
Re-operation	906 patients in 15 studies	8.03% (5.97–12.72)	*I*^2^: 60.3% (30.1–77.4)	HA: 6.24% (3.92–9.78) PISA: 17.24 (9.38–29.55) HR: 4.29 (2.06–8.73)	<0.001
Forward elevation	265 patients in 7 studies	43.9 degrees (36.7–51.2)	*I*^2^: 29.3% (0–69.7) PI: 29.37–58.49	HA: 43.9 (30.2–57.3) PISA: 43.4 (21.3–65.6)	0.954
External rotation	265 patients in 7 studies	24.1 degrees (18.4–29.9)	*I*^2^: 50.8% (0–79.1) PI: 13.11–35.11	HA: 26.5 (25.4–27.6) PISA: 19.6 (−6.3 to 45.5)	0.252
Constant Score	355 patients in 9 studies	34.7 (29.4–40.1)	*I*^2^: 71% (42.5–85.3) PI: 20.9–48.59	HA: 36.1 (28.0–44.2) PISA: 33.0 (25.7–40.4)	0.397
Subjective Shoulder Value	185 patients in 5 studies	40.6 (33.4–47.8)	*I*^2^: 54.2% (0–79.1) PI: 25.88–55.32	HA: 41.7 (24.4–59.1) PISA: 38.7 (20.8–56.6)	0.473

CI: confidence interval; HA: hemiarthroplasty; PISA: pyrocarbon interposition shoulder arthroplasty; HR: hemi-resurfacing; PI: prediction interval.

### Reoperation rate

The overall pooled reoperation rate was 8.03% (95% CI 5%–12.7%) across fifteen studies. When stratified by type of implant, statistically significant higher reoperation rates were observed in patients treated with PISA (17.2%; 95% CI 9.4%–29.6%) when compared with hemiarthroplasty (6.2%, 95% CI 3.9%–9.8%) and hemi-resurfacing (4.3% 95% CI 2.1%–8.7%) (*p* < 0.001). The most common reasons for reoperation were post-operative rotator cuff insufficiency (19%), painful glenoid erosion (17.5%) and infection (9.5%). Other reasons for reoperation included greater tuberosity fracture (6.3%), implant failure with head breakage (6.3%), iatrogenic instability (7.9%) and stiffness (3.2%). Reason for reoperation was unspecified in 7.9% of cases. Reoperation procedures included revision to reverse TSA (rTSA), revision to TSA, irrigation and debridement, implant exchange and arthrolysis. Pooled reoperation data is provided in Table 3. Reasons for reoperation for each included study are summarized in Table 4.

**Table 4.** Reoperation rate.

Author & year	Procedure	Reoperation	*N*	Rate	Reoperation reason (procedure)
Barret 2019	PISA	8	58	13.80%	4 GT fracture (4 rTSA), 2 painful glenoid wear (4 rTSA), 2 stiffness (2 arthrolysis)
Barret 2021	HA	4	30	13.30%	2 infection, 1 false humeral stem path, 1 superior head migration with rotator cuff damage
Caughey 2023	HA	2	56	3.60%	1 infection (rTSA), 1 rotator cuff tear (rTSA)
Cointat 2022	HA	5	64	7.80%	1 painful glenoid erosion (TSA), 4 rotator cuff deficiency (4 rTSA)
Garret 2022	PISA	11	67	16.40%	2 posterior subluxation (1 TSA, 1 glenoid revision), 2 inferior GH impingement (1 TSA, 1 osteophyte removal), 2 rotator cuff tear (2 rTSA), 3 persistent glenoid pain (3 TSA), 1 supraspinatus ossification (arthrolysis), 1 painful shoulder with distal subsidence (rTSA)
Garret 2019	HA	3	65	4.60%	2 painful glenoid (1 rTSA, 1 exchange for metal head), 1 rotator cuff tear (rTSA)
Gao 2023	HA	5	159	3.10%	3 overstuffed glenoid (3 revision to smaller humeral head implant), 2 rotator cuff tear (2 rTSA)
Hirakawa 2021	PISA	5	10	50%	5 unspecified (rTSA)
Hudek 2017	PISA	1	10	10%	1 unspecified (arthroscopic debridement)
Kany 2021	HA	4	24	16.70%	1 deep infection (lavage), 1 instability (rTSA), 2 glenoid wear (2 implant exchange)
Khoriarti 2023	HA	7	159	4.4%	2 instability, 1 glenoid wear, 1 infection, 1 rotator cuff insufficiency, 1 pain
Kleim 2021	HA	0	21	0	n/a
McBride 2022	HR	7	163	4.30%	2 pain, 4 implant failure with head breakage, 1 infection
Novi 2022	HA	0	4	0	n/a
Tsitlakidis 2021	HA	1	16	6.30%	1 periprosthetic fracture

HA: hemiarthroplasty; HR: hemi-resurfacing; GH: glenohumeral; GT: greater tuberosity; PISA: pyrocarbon interposition arthroplasty; rTSA: reverse total shoulder arthroplasty.

## Discussion

The use of pyrocarbon as a bearing surface in shoulder hemiarthroplasty and more recently PISA has gained traction in Europe and Australia. In comparison, its use in North America is relatively novel. When introducing new technologies it is important to follow a stepwise evidence-based approach to ensure safe and efficacious treatments remain the cornerstone of operative management.^39^ Pyrocarbon has a modulus of elasticity which more closely resembles bone than other metallic shoulder arthroplasty bearing surfaces such as cobalt chromium.^40^ Pyrocarbon surfaces also demonstrate a strong affinity to phospholipids which reduces friction between articulating pyrocarbon-coated implants and native glenoid cartilage.^10^ These advantageous properties are theorized to reduce the potential for glenoid wear, which is particularly problematic in younger patients who are treated with traditional hemiarthroplasty implants. *In vitro* studies comparing cobalt chromium and pyrocarbon implants have shown significantly reduced articular surface wear with pyrocarbon implants. Klawitter et al. simulated shoulder hemiarthroplasty wear conditions with cyclical glenoid loading at 760 N with pyrocarbon and cobalt chromium implants. Complete articular surface wear occurred at 32,000 cycles for cobalt chromium implants versus 5,000,000 cycles for pyrocarbon implants. Bone volume loss and implant surface roughness were 30× greater with cobalt chromium implants versus pyrocarbon implants, further outlining the potential benefits of a pyrocarbon bearing surface.^40^

Young patients place greater loads on shoulder arthroplasties due to higher activity levels and occupational demands. As a result, implant survivorship is a key consideration in this patient population, necessitating durable, wear-resistant bearing surfaces in modern implants. The studies included in the present review, evaluated a cohort of young patients with a median age of 53 years, a relatively young patient population. The pooled revision rate in our analysis was 8.03% at a median follow-up period of 38 months. For studies using pyrocarbon hemiarthroplasty components, the revision rate was 6.24%. This represents a lower revision rate when compared with available literature evaluating conventional shoulder hemiarthroplasty in similar young patient populations. A systematic review from Fonte et al.^41^ evaluated outcomes of shoulder hemiarthroplasty in young patients. Ten studies with a total of 341 patients and an average age of 47.03 years were reported. The revision rates of the included studies were much higher than the present study ranging from 11.11% to 30%.^41^ Three studies included in the present review compared pyrocarbon implants with traditional metallic materials. Kany et al.^25^ found a higher revision rate in a sample of 10 metallic implants (30%) when compared to pyrocarbon implants (17%).^25^ Similarly, McBride et al.^29^ reported high revision rates with 163 metallic hemi-resurfacing (17.1%) and 67 metallic stemmed hemiarthroplasties (17.5%) when compared to pyrocarbon hemi-resurfacing (8.9%).^29^ Khoriati et al.^38^ utilized Australian Joint Registry data to compare revision rates of metallic head hemiarthroplasty, stemmed pyrocarbon hemiarthroplasty and hemi-resurfacing implants in patients <55 years old. At four years, metallic stemmed implants demonstrated higher revision rates (16.7%) when compared with stemmed pyrocarbon (8.9%) and hemi-resurfacing procedures (8.1%).^38^

The most commonly reported reasons for reoperation in the present review included rotator cuff insufficiency (19%), painful glenoid erosion (17.5%) and infection (9.5%). A large retrospective cohort study from Dillon et al.^42^ evaluated revision risk in 504 patients aged <59 years old who were treated with hemiarthroplasty, rTSA, TSA and humeral head resurfacing. This cohort reported a significantly higher proportion of revision for glenoid wear (70.4%) when compared to the present study.^42^ These results may support a decreased risk of glenoid wear with pyrocarbon implants as noted in the present study. Although our review is limited by a higher rate of unspecified reason for reoperation (7.9%). Of note, catastrophic implant failure with head breakage was reported in 6.3% of reoperation cases in the present review.

While implant longevity is a primary concern in young active patients, optimizing patient satisfaction for these high-demand individuals represents another key factor in improving treatment outcomes. Functional measures reported in the present study included range of motion, Constant Score and SSV. Mean range of motion improvement in the present study was 43.9 degrees in forward elevation and 24.1 degrees in external rotation. In comparison, Fonte et al.^41^ reported range of motion improvements of 20.9 degrees and 27.9 degrees in forward elevation and external rotation, respectively, for hemiarthroplasty patients. Forward elevation improvements noted in the present study were greater by comparison. The review from Fonte et al.^41^ noted similar improvement in forward elevation in patients treated with rTSA (48.6 degrees) with expectedly quite lower improvements in external rotation (11.4 degrees) when compared to the present review.^41^ In our review, the average improvement in Constant Score was 34.7, while SSV improvement was 40.6. In comparison, Fonte et al.^41^ reported an average improvement in Constant Score of 29.6 in hemiarthroplasty patients. The Constant Score improvement observed in the present review was in keeping with those reported for TSA (33.8) and rTSA patients (34.6) in the review by Fonte et al.^41^ These findings suggest acceptable functional improvements with pyrocarbon implants, although long-term follow-up and comparison studies are required to further evaluate their potential benefit.

Our study has notable limitations. The follow-up period of included studies was relatively short. Therefore, it is possible that future reoperation events in this treatment population have not been adequately captured in the pooled sample. As well, we acknowledge the risk of bias in the included studies was substantial with only two studies being rated as low risk. Lastly, a large amount of statistical heterogeneity was observed in our analyses with moderate heterogeneity noted in our evaluation of reoperation rate, external rotation and SSV. Substantial heterogeneity was observed in pooled Constant Score estimate. Further high-quality research with long-term follow-up is needed to evaluate the use of pyrocarbon implants in shoulder arthroplasty.

## Conclusion

Pyrocarbon shoulder implants, in particular hemiarthroplasty and hemi-resurfacing components, demonstrated notable improvements in functional outcomes with acceptable reoperation rates at early- to mid-term follow-up. As such, they represent a promising alternative to traditional bearing surfaces, especially in the young, active patient with GHO. However, further well-designed prospective studies with long-term follow-up are required to verify the safety and efficacy of pyrocarbon bearing surfaces in shoulder arthroplasty.
